# The therapeutic properties of resminostat for hepatocellular carcinoma

**DOI:** 10.18632/oncoscience.420

**Published:** 2018-06-23

**Authors:** Jun Zhao, Steven G. Gray, Martin Wabitsch, Catherine M. Greene, Matthew W. Lawless

**Affiliations:** ^1^ Experimental Medicine, UCD School of Medicine and Medical Science, Mater Misericordiae University Hospital, Dublin, Ireland; ^2^ Department of Clinical Medicine, Trinity Centre for Health Sciences, Trinity Translational Medicine Institute, St. James's Hospital & Trinity College, Dublin, Ireland; ^3^ Division of Paediatric Endocrinology and Diabetes, Department of Paediatrics, University of Ulm, Ulm, Germany; ^4^ Clinical Microbiology, Royal College of Surgeons in Ireland, Beaumont Hospital, Dublin, Ireland

**Keywords:** HDAC inhibitor resminostat, HCC, adipocytes, HSP90 inhibitor 17-AAG, ER stress/UPR

## Abstract

Hepatocellular carcinoma (HCC) is the most common form of primary liver cancer with increases in new cases being reported annually. Histopathologists have identified hepatic steatosis as a characteristic of a broad range of chronic liver diseases that are associated with the onset and development of HCC. In this context, epigenetic modifications may serve as precancerous factors predisposing normal cells to the initiation of carcinogenesis. This study demonstrated that hepatic tumorigenesis and differentiated adipocytes may modulate both global histone deacetylase (HDAC) expression and specific class I HDAC genes in the tumour microenvironment. The novel class I HDAC inhibitor Resminostat was shown to reduce the proliferation of HCC cells along with its specificity in targeting class I HDACs and oncogenes. The combined effect of Resminostat with several pharmaceutical agents such as Sorafenib, Cisplatin and Doxorubicin was also demonstrated. The inhibition of heat shock protein 90 (HSP90) has been demonstrated as a potential therapeutic option for HCC. In line with this, the specific HSP90 inhibitor 17-(allylamino)-17-demethoxygeldanamycin (17-AAG) was selected and it was found that the combination of Resminostat and 17-AAG may provide a “smart” clinical strategy for HCC patients by targeting cellular communication within the tumour microenvironment. This study provides an insight into the use of Resminostat as an epigenetic based therapeutic for HCC along with other pharmaceutical options, in particular by targeting the cell-to-cell communication that occurs between hepatoma and adipocytes.

## INTRODUCTION

The concept of epigenetics was first introduced as an important element in embryonic development by Conrad Hal Waddington in 1942 [1]. Recent advances have elucidated epigenetic functions in many biological processes and global alterations in the epigenetic landscape have been proposed as a hallmark of cancer [2, 3]. In fact, epigenetic mechanisms are fundamental in cellular homeostasis and include a broad range of activities such as DNA methylation, histone modification, chromatin structure remodelling and non-coding RNA interactions [4]. While the onset and development of hepatocellular carcinoma (HCC) have been attributed to several factors at both cellular and physiological levels, epigenetic alterations are receiving an increased attention in the investigation of the pathogenesis and pharmaceutical targeting of liver cancer due to their nature of reversibility.

To-date, several independent genome-wide methylation profiling studies have revealed an aberrant DNA methylation pattern in HCC tumour samples compared to adjacent normal liver tissue suggesting potential diagnostic and prognostic epigenetic signatures for liver cancer patients [5-7]. At the cellular level, DNA strands are coiled around the histone tails to form chromatin. Histone modifications such as acetylation/deacetylation and methylation on histone tails allow the activation or inhibition of specific genes, the aberrant expression of which are associated with HCC onset and progression. Indeed, a recent cohort study carried out in 170 HCC patients has shown that class I HDACs (HDAC 1, 2 and 3) were highly expressed in HCC tumour tissues compared to adjacent normal tissues [8]. In addition, overexpression of HDAC1 and 2 is strongly associated with poor prognosis and higher mortality rates in HCC patients [9-11]. Histone acetylation and deacetylation are sub-types of epigenetic mechanisms balancing the expression of critical genes involved in tumorigenesis such as proliferation, cell-cycle regulation and apoptosis. Histone acetylation is regulated by histone acetyl transferases (HATs) loosening the chromatin structure, thus allowing gene transcription. In contrast, HDACs generate a non-permissive chromatin structure inhibiting the expression of their target genes. Moreover, epigenetic targeted therapy such as HDAC inhibition has become one of the most promising anti-cancer strategies due to the reversibility of aberrant epigenetic changes during oncogenesis [12]. Currently, Sorafenib is the only Food and Drug Administration (FDA) approved pharmaceutical agent for advanced HCC patients, giving them a 3 month extended survival time. Resminostat is a novel oral pan-HDAC inhibitor specifically targeting class I HDACs that has attracted attention from its use in a phase II clinical trial for HCC patients. The recent SHELTER study examining Resminostat in combination with Sorafenib showed a promising 8 month survival time for advanced HCC patients [13].

We have previously described that close interactions between hepatic tumorigenesis and adipocytes are associated with a series of negative cellular events involving, angiogenesis, inflammation, insulin homeostasis and immunomodulation that may give rise to hepatocarcinogenesis [14]. Here we investigate epigenetic alterations in the tumour microenvironment involving cell-to-cell communication between HCC and adipocytes.

## RESULTS

### Effects of conditioned medium on HDACs in human adipocytes and liver cancer cells

**Figure 1:**
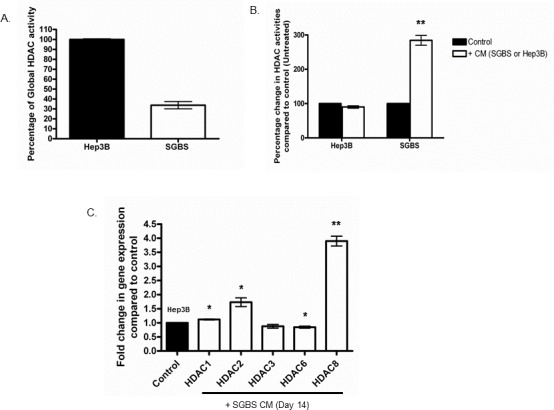
Effects of CM on HDACs in human adipocytes and liver cancer cells Liver cancer cell line (Hep3B) and differentiated human adipocyte SGBS were cultured alone or treated with 30% of the CM from each other for 24 hours (n = 3). Their global HDAC activities were measured with FluoroFire HDAC Activity Assay Kit. **(A)** Global HDAC activity in Hep3B and differentiated SGBS cells. **(B)** Global HDAC activities in Hep3B and differentiated SGBS cells treated with CM from each other. **(C)** The mRNA expression levels of class I HDACs (HDAC 1, 2, 3 and 8) and class II HDAC (HDAC6) were measured in Hep3B cells treated with 30% differentiated SGBS CM by RT-PCR and presented as fold change compared to control (untreated cells).

A recent cohort study carried out in 170 HCC patients has shown that class I HDACs (HDAC 1, 2 and 3) were highly expressed in HCC tumour tissues compared to adjacent normal tissues [8]. We examined the global HDAC activities in liver cancer Hep3B cells and differentiated human adipocytes SGBS cells. We found that differentiated human adipocytes had 62.33% less HDAC activity compared to HCC cells (Figure 1A). We then measured the global HDAC alterations in (i) Hep3B cells treated with 30% conditioned medium (CM) from differentiated SGBS cells and (ii) differentiated SGBS cells treated with 30% CM from Hep3B cells. Surprisingly, CM from Hep3B cells increased the global HDAC activities of differentiated SGBS cells by nearly 3-fold (Figure 1B). While CM from differentiated Simpson-Golabi-Behmel syndrome (SGBS) cells did not change the global HDAC activities of Hep3B cells, it did however specifically increase the expression of the class I HDACs (HDAC 1, 2 and 8) at the mRNA level (Figure 1C). Differentiated SGBS CM also decreased the gene expression of HDAC6 in Hep3B cells at the mRNA level (Figure 1C).

### Class I HDAC inhibitor Resminostat inhibits the proliferation of HCC cells

**Figure 2:**
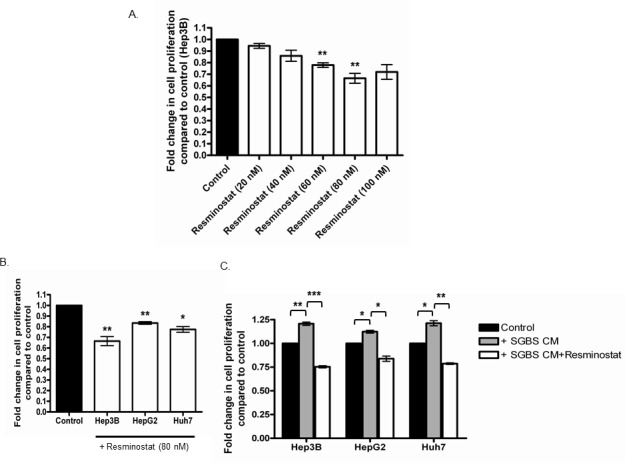
The anti-proliferative effect of Class I HDAC inhibitor Remininostat on HCC cells with and without SGBS CM **(A)** Hep3B cells were treated with Resminostat in a dose range from 20 nM to 100 nM for 24 hours and their proliferation was measured with FluoroFire-Blue ProViaTox assay and presented as fold change compared to control (untreated cells) (n = 3). **(B)** Hep3B, HepG2 and Huh7 cells were treated with Resminostat (80 nM) and their proliferation were measured and presented as fold change compared to control (untreated cells). **(C)** The proliferation of Hep3B, HepG2 and Huh7 cells treated with SGBS CM (30%) alone or SGBS CM (30%) in combination with Resminostat (80 nM) for 24 hours was measured and presented as fold change compared to control (untreated cells) or between treatments.

We selected a class I HDAC inhibitor Resminostat as a potential pharmaceutical agent against HCC. Resminostat underwent a phase II clinical trial exhibiting promising clinical outcomes in both safety and survival in advanced HCC patients. Hep3B cells were treated with Resminostat in a dose range of 20 nM, 40 nM, 60 nM, 80 nM and 100 nM for 24 hours. Resminostat showed an anti-proliferative effect on Hep3B cells in a dose dependent manner (Figure 2A). At 80 nM Resminostat reduced the proliferation of three liver cancer cell lines Hep3B, HepG2 and Huh7 after 24 hours treatment (Figure 2B). This effect was also evident in all cells co-treated with 30% SGBS CM (Figure 2C).

### Resminostat reduces global HDAC activities and suppresses HDAC1, 2 and 3 expressions in HCC cells

**Figure 3:**
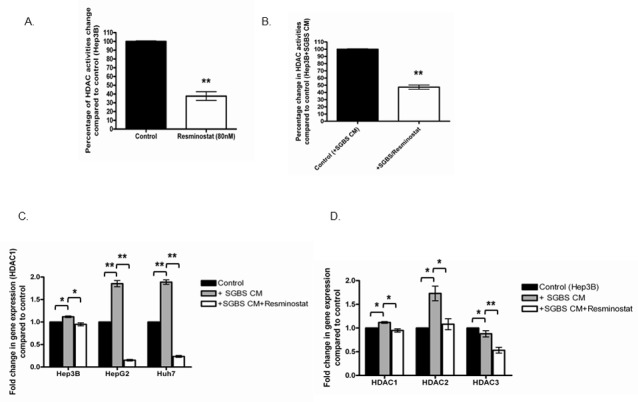
Resminostat suppressed global and specific class I HDAC genes **(A)** Hep3B cells were treated with Resminostat (80 nM) for 24 hours and the global HDAC activity was measured compared with control (untreated cells) (n = 3). **(B)** Global HDAC activity in Hep3B cells treated with SGBS CM (30%) and Resminostat (80 nM) was analysed compared with Hep3B cells treated with SGBS CM (30%). **(C)** The RNA levels of HDAC1 in Hep3B, HepG2 and Huh7 cells treated with either SGBS CM (30%) or SGBS CM (30%) with Resminostat (80 nM) were quantified and presented as fold change compared to control (untreated cells) or between treatments (n = 3). **(D)** Hep3B cells were treated with either SGBS CM (30%) or SGBS CM (30%) with Resminostat (80 nM). The RNA levels of HDAC 1, HDAC2 and HDAC3 were measured and presented as fold change compared to control (untreated cells) or between treatments.

We tested the effect of Resminostat against global HDAC activities and expression levels of specific class I HDACs. Figure 3 shows that Resminostat (80 nM) decreased 62.33% of the global HDAC activities in Hep3B cells after 24 hours (Figure 3A). In addition, Resminostat (80 nM) reduced nearly 52.67% of the global HDAC activities in Hep3B cells treated with 30% SGBS CM (Figure 3B). Resminostat (80 nM) reduced the gene expression of HDAC1 at the mRNA levels in Hep3B, HepG2 and Huh7 cells treated with SGBS CM (Figure 3C). Moreover, Resminostat (80 nM) suppressed the expression of HDAC1, 2 and 3 at the mRNA levels in Hep3B cells treated with SGBS CM after 24 hours (Figure 3D).

### The anti-proliferative effect of Resminostat is associated with increased Caspase activities and alteration of BCL-x and cell cycle genes

**Figure 4:**
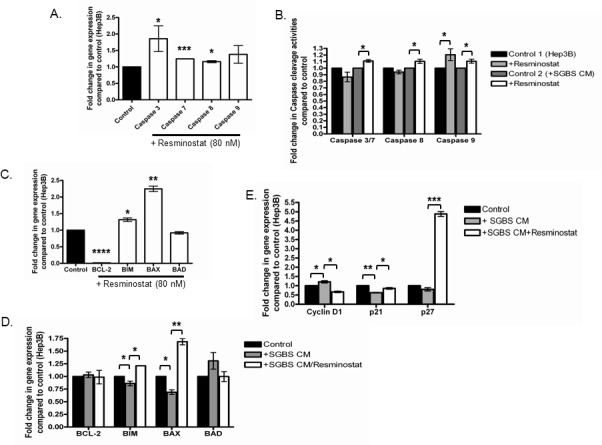
The anti-proliferative effect of Resminostat is associated with increased Caspase activities and the alteration of BCL-x and cell cycle family genes **(A)** The RNA levels of Caspase 3, Caspase 7, Caspase 8 and Caspase 9 were measured in Hep3B cells treated with 30% SGBS CM for 24 hours and presented as fold change compared with control (untreated cells) (n = 3). **(B)** Hep3B cells were treated with (i) Resminostat (80 nM) (ii) SGBS CM (30%) with and without Resminostat (80 nM) for 24 hours and the cleavage activity of Caspase 3/7, Caspase 8 and Caspase 9 was measured compared with controls or between treatments (n = 3). **(C)** The RNA levels of BCL-2, BIM, BAX and BAD in Hep3B cells treated with Resminostat (80 nM) for 24 hours. **(D)** The RNA levels of BCL-2, BIM, BAX and BAD in Hep3B cells treated with either SGBS CM (30%) or SGBS CM (30%) with Resminostat (80 nM) for 24 hours. **(E)** RNA expression levels of Cyclin D1, p21 and p27 in Hep3B cells treated with either SGBS CM (30%) or SGBS CM (30%) with Resminostat (80 nM).

We next investigated the potential mechanisms in anti-proliferative effect of Resminostat on HCC. Hep3B cells were treated with Resminostat alone or in combination with SGBS CM for 24 hours. Caspase 3, 7, 8 and 9 gene expressions were examined as well as their protein cleavage activities following treatments. Figure 4A shows that Resminostat increased the mRNA levels of Caspase 3, 7 and 8 in Hep3B cells. Resminostat only increased protein cleavage activity of Caspases 9 in Hep3B cells (Figure 4B). Interestingly, Resminostat (80 nM) elevated the protein cleavage activities of Caspase 3/7, 8 and 9 in Hep3B cells when they were treated with SGBS CM (Figure 4B). We then analysed the mRNA expression levels of BCL-x family members and cell cycle regulation genes in HCC cells following Resminostat treatment. Resminostat notably downregulated the expression levels of BCL-2 and upregulated BIM and BAX (Figure 4C). In addition, Resminostat (80 nM) increased the levels of BIM and BAX mRNA in Hep3B cells treated with SGBS CM (Figure 4D). There was no effect on BAD expression. Moreover, Resminostat (80 nM) decreased Cyclin D1 and increased p21 and p27 mRNA expression in Hep3B cells treated with SGBS CM (Figure 4E).

### Resminostat targets a panel of tumorigeneic genes in HCC associated with excessive adipocytes

**Figure 5:**
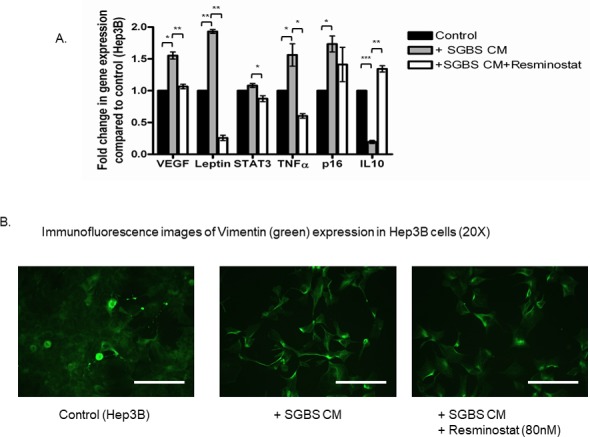
Resminostat reduced metastatic progression in HCC under SGBS CM treatment **(A)** Hep3B cells were cultured in 30% SGBS CM alone or in combination with Resminostat (80 nM) for 24 hours and their total RNAs were extracted for gene analysis with RT-PCR. A panel of oncogenes including VEGF, leptin, STAT3, TNFα, p16 and IL-10 were measured at the RNA level (n = 3). **(B)** Hep3B cells were cultured in 30% SGBS CM alone or in combination with Resminostat (80 nM) for 24 hours. Cells were then fixed and stained with primary antibody (anti-Vimentin mouse monoclonal) and secondary antibody (anti-mouse green immunofluorescence). Scale bar = 20 μm.

This study examined a panel of tumorigeneic genes that may be targeted in HCC under the co-treatment of SGBS CM. Figure 5 shows that Resminostat (80 nM) reduced the mRNA levels of VEGF, leptin, STAT3, TNFα and increased expression levels of IL-10 in HCC cells treated with adipocyte CM after 24 hours (Figure 5A). We also performed immunofluorescence imaging of Vimentin (green) protein expression in Hep3B cells treated with SGBS CM and Resminostat after 24 hours compared to untreated cells as control. SGBS CM markedly enhanced the Vimentin protein expression levels in Hep3B cells and this appeared attenuated when treated with Resminostat (Figure 5B).

### Resminostat enhanced the anti-proliferative effect of other pharmacological agents against HCC

**Figure 6:**
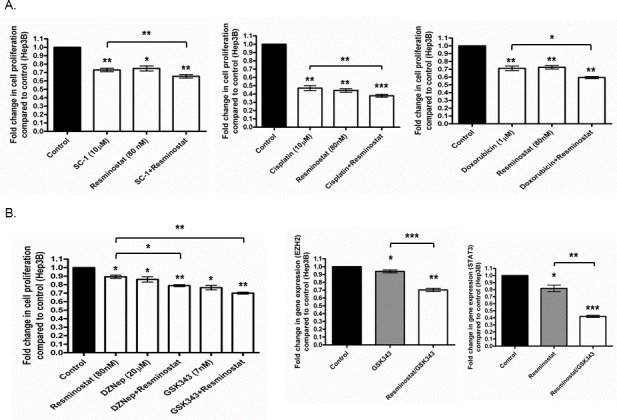
Resminostat enhanced the anti-proliferative effects of pharmaceutical agents for HCC **(A)** Hep3B cells were treated with SC-1 (10 μM) (a derivative of FDA approved Sorafenic), Cisplatin (10 μM) and Doxorubicin (1 μM) alone or in combination with Resminostat (80 nM) for 24 hours and their proliferation was measured with FluoroFire-Blue ProViaTox assay and presented as fold change compared to control (untreated cells) and between treatments (n = 3). **(B)** Hep3B cells were treated with novel demethylating agents DZNep (20 μM), GSK343 (7 nM) alone or in combination with Resminostat (80 nM) for 24 hours and their proliferation was measured and presented as fold change compared to control (untreated cells) and between treatments. RNA expression levels of EZH2 and STAT3 were analysed between GSK343 (7 nM) and GSK343 (7 nM)/Resminostat (80 nM) combination treatments.

Recent studies have shown that Resminostat had additive or synergistic activities in combination with other novel pharmaceutical agents and conventional chemotherapeutic agents for HCC [15]. We selected the FDA approved Sorafenib derivative SC-1 and cytotoxic chemo-drugs (Cisplatin and Doxorubicin) while testing them in combination with Resminostat for their anti-proliferative effects against HCC (Dose optimisation see Supplementary Figure 1). Figure 6 shows that SC-1 (10 μM), Cisplatin (10 μM) and Doxorubicin (1 μM) reduced the proliferation of Hep3B cells and Resminostat (80 nM) further reduced the proliferation of HCC cells by 10.27%, 19.57% and 16.67% respectively (Figure 6A).

Chromatin modifiers, in particular the polycomb-repressive complexes 2 subunit IEnhancer of zeste homolog 2 (EZH2), have been reported in many studies to be overexpressed in HCC samples and this is often associated with poor prognosis in liver cancer patients [16-18]. We selected novel pharmaceutical agents DZNep and GSK343 that specifically target the histone methyltransferase EZH2 to examine their anti-HCC effects. Both DZNep (20 μM) and GSK343 (7 nM) reduced the proliferation of Hep3B cells after 24 hours and the combinations of Resminostat/DZNep and Resminostat/GSK343 had further reduced proliferation by 11.76% and 21.61% respectively (Figure 6B). In addition, Resminostat (80 nM) enhanced the downregulation of the gene expression levels of EZH2 and STAT3 in Hep3B cells with GSK343 treatment (Figure 6B).

### 17-AAG enhanced the anti-proliferative effect of Resminostat under SGBS CM treatment

**Figure 7:**
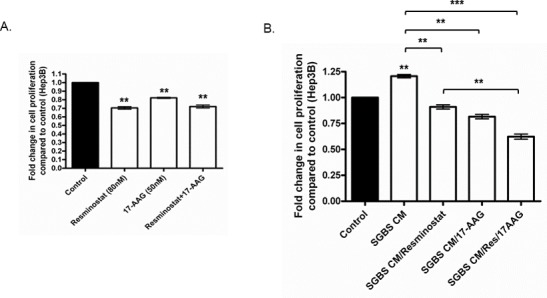
Resminostat and 17-AAG had combined anti-proliferative effect against HCC even when challenged with SGBS CM **(A)** The proliferation of Hep3B cells treated with Resminostat (80 nM), 17-AAG (50 nM) and their combination was measured after 24 hours with FluoroFire-Blue ProViaTox assay (n = 3). **(B)** The proliferation was measured in Hep3B cells treated with SGBS CM and either Resminostat, 17-AAG or their combination after 24 hours.

We previously proposed that Endoplasmic Reticulum (ER) stress mediator heat shock protein 90 (HSP90) may play an important role in a HCC-adipocyte axis [14]. In this context, the HSP90 inhibitor, 17-(allylamino)-17-demethoxygeldanamycin (17-AAG), was used to examine its potential therapeutic effects in combination with Resminostat. Figure 7A shows that both Resminostat (80 nM) and 17-AAG (50 nM) reduced the proliferation of HCC cells, but their combination did not further improve the anti-proliferative effect. Interestingly, 17-AAG (50 nM) was shown to have a combined effect in reducing the proliferation of HCC cells by 31.47% with Resminostat (80 nM) under SGBS CM treatment (Figure 7B).

### Combined effects of Resminostat/17-AAG on class I HDACs and oncogenic genes in HCC

**Figure 8:**
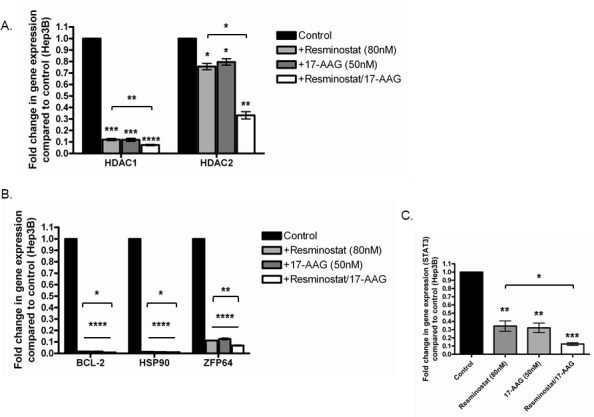
Resminostat and 17-AAG had enhanced anti-HCC effects compared to single treatment **(A)** The RNA levels of HDAC1 and HDAC2 were measured in Hep3B cells treated with Resminostat (80 nM), 17-AAG (50 nM) or their combination for 24 hours (n = 3). **(B)** A panel of genes including BCL-2, HSP90 and ZFP64 were analysed at RNA levels in Hep3B cells treated with Resminostat (80 nM), 17-AAG (50 nM) and their combination. **(C)** The RNA levels of STAT3 were measured in Hep3B cells treated with Resminostat (80 nM), 17-AAG (50 nM) or their combination for 24 hours.

Hep3B cells were treated with either Resminostat (80 nM), 17-AAG (50 nM) or their combination for 24 hours and a panel of genes including class I HDACs and oncogenic genes were analysed. The data revealed that the combination of Resminostat (80 nM) and 17-AAG (50 nM) further downregulated HDAC1 and HDAC2 in Hep3B cells compared to either Resminostat (80 nM) or 17-AAG (50 nM) treatment (Figure 8A). Moreover, the combination of Resminostat (80 nM) and 17-AAG (50 nM) was demonstrated to have enhanced an inhibitory effect on BCL-2, HSP90, ZFP64 and STAT3 mRNA levels in Hep3B cells (Figure 8B and Figure 8C).

### The anti-inflammatory function of 17-AAG in HCC associated with SGBS and Resminostat treatment

**Figure 9:**
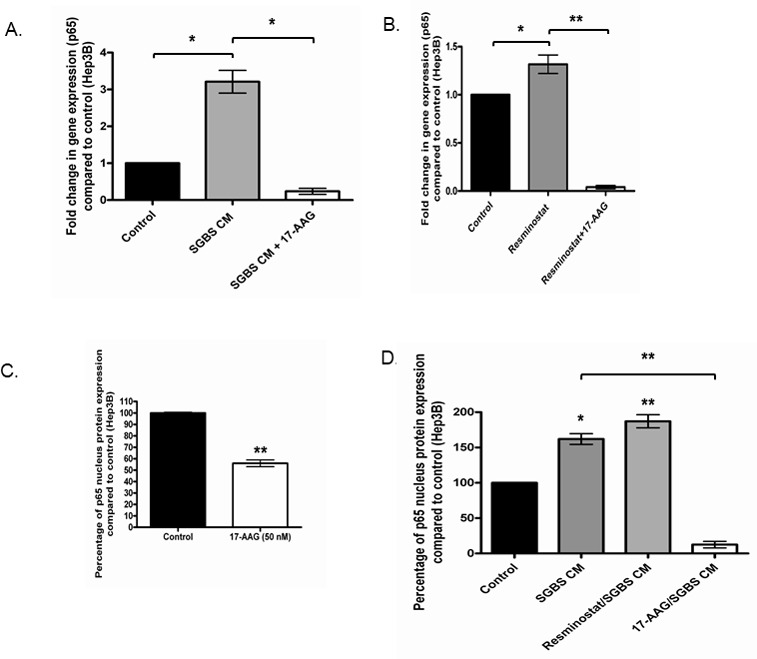
17-AAG inhibited the pro-inflammatory gene NFκB (p65) at both RNA and protein levels **(A)** The RNA levels of p65 were measured in Hep3B cells treated with either SGBS CM (30%) or SGBS CM with 17-AAG (50 nM) for 24 hours (n = 3). **(B)** The RNA levels of p65 were analysed in Hep3B cells treated with either Resminostat (80 nM) or Resminostat with 17-AAG (50 nM) for 24 hours. **(C)** The nuclear protein levels of p65 were quantified in Hep3B cell treated with 17-AAG (50 nM) compared with untreated cells (n = 3). **(D)** The nuclear protein levels of p65 were measured in Hep3B cells treated with (i) SGBS CM (30%) (ii) SGBS CM (30%) with Resminostat (80 nM) and (iii) SGBS CM (30%) with 17-AAG (50 nM) for 24 hours and compared with untreated cells.

Recent studies demonstrated that HDACis may switch on oncogenes such as the pro-inflammatory NFκB subunit p65 while suppressing a broad range of other tumorigenic molecules [19]. Figure 9A shows that the HSP90i 17-AAG reduced p65 RNA expression levels in HCC cells under SGBS CM. Moreover, 17-AAG almost completely diminished the p65 RNA expression that was elevated by Resminostat in Hep3B cells (Figure 9B). Furthermore, 17-AAG notably reduced the nuclear protein expression levels of p65 in Hep3B cells alone or treated with SGBS CM (Figure 9C and Figure 9D).

## DISCUSSION

We previously described that excessive adipocytes may be associated with, angiogenesis, inflammation, insulin resistance and immunomodulation in hepatocarcinogenesis. Epigenetic mechanisms including DNA methylation, histone modifications and non-coding RNA interactions are crucial for normal cellular functioning through regulating the activation and inhibition of specific genes. In fact, epigenetic alteration has been suggested as a surrogate of genetic mutations which indicates that aberrant epigenetic modifications may serve as an essential precancerous factor predisposing normal cells to the initiation of carcinogenesis [20]. Unlike genetic mutations, epigenetic alterations are reversible making them one of the most promising therapeutic targets in current cancer treatment strategies. Indeed, recent studies have shown that hepatic steatosis can activate tumorigenic signalling pathways through the altered expression of HDACs in HCC patients associated with Non-alcoholic fatty liver disease (NAFLD) [21]. In this context, we examined the global HDAC activity in both Hep3B and differentiated SGBS cells and it was found that differentiated adipocytes had 3-fold less global HDAC activity than HCC cells. Interestingly, CM from Hep3B cells significantly increased the global HDAC activity in differentiated SGBS cells. We did not observe any change of global HDAC activity in Hep3B cells cultured with CM from differentiated SGBS cells. This may be due to the high basal levels of global HDAC activity in Hep3B cells and subtle changes could not be detected by this assay. Importantly, we analysed the gene expression of class I HDACs in Hep3B cells cultured with CM from differentiated SGBS cells and found that this increased the expression of the class I HDACs HDAC 1, 2 and 8 suggesting that differentiated adipocytes may manipulate specific HDAC expression rather than global HDAC activity in liver cancer. It is worth noting that SGBS CM reduced the gene expression levels of HDAC 6 in Hep3B cells. While the exact role of HDAC 6 in HCC remains controversial, further studies will be required [22 – 24].

We next selected a specific class I HDAC inhibitor Resminostat as the potential pharmaceutical agent to investigate its anti-cancer effects against HCC. Importantly, HDAC inhibitors have been identified to reverse transformed malignant cells making them ideal candidates as therapeutic agents in cancer treatment for HCC [25]. Resminostat is a pan-HDACi which has undergone a phase II clinical trial. In the recent multicentered phase II SHELTER study, patients with advanced HCC were given Resminostat in a 5-day-on and 9-day-off pattern along with Sorafenib administration. The combination of Resminostat and Sorafenib achieved an unexpected 70% progression free survival at 12 weeks and the overall survival of HCC patients were improved from 3 to 8 months [13]. In this study, we first validated the optimal dose of Resminostat by treating Hep3B cells at doses ranging from 20 nM to 100 nM for 24 hours and found that Resminostat reached its optimal anti-proliferative effect against Hep3B cells at 80 nM after 24 hours and no further anti-proliferative effect was found at 100 nM treatment. Moreover, we showed that at 80 nM Resminostat significantly reduced the proliferation of all three liver cancer cell lines Hep3B, HepG2 and Huh7 even under treatment with the SGBS CM. Importantly, Resminostat not only reduced the global HDAC activity of Hep3B cells by nearly 62.33%, but it also decreased 52.67% of the global HDAC activity in Hep3B cells cultured with SGBS CM. In addition, Resminostat significantly downregulated the expression of class I HDACs (HDAC1, 2 and 3) in Hep3B, HepG2 and Huh7 cells cultured with SGBS CM.

HDAC1 and 2 are often over-expressed in human HCC samples and have been shown to directly target tumour suppressor gene p21 [9, 10, 26, 27]. In this regard, we investigated the molecular mechanisms of the anti-proliferative effects of Resminostat against HCC on apoptotic, cell cycle and BCL family signalling pathways. The mRNA levels of Caspase 3, 7 and 8 in Hep3B cells were elevated by Resminostat. In addition, the cleavage activity of Caspase 9 protein was significantly increased by Resminostat. This is supported by recent studies showing that HDAC is such as Valproate acid sodium, Vorinostat and Romidepsin selectively induced apoptosis of tumour cells including HCC via an intrinsic apoptosis pathway rather than an extrinsic apoptosis pathway [28]. Interestingly, Resminostat activated both intrinsic and extrinsic apoptosis pathways by inducing Caspase 8, 9 and 3/7 protein cleavage activities in Hep3B cells cultured with differentiated adipocytes CM. Moreover, Resminostat notably downregulated the gene expression of BCL-2 and upregulated pro-apoptotic genes BIM and BAX in Hep3B cells even when challenged with SGBS CM. Furthermore, Cyclin D1 RNA expression was reduced and associated with the upregulation of p21 and p27 in Hep3B cells cultured with SGBS CM. These data are supported by several studies showing that HDACis can specifically target the BCL family members and cell cycle genes including Cyclin D1 and p21. These findings suggest that Resminostat may simultaneously target several apoptotic signalling pathways to induce an anti-proliferative effect against HCC.

HCC associated with excessive adipocytes is characterised by increased production of oncogenes which can exacerbate tumorigenesis. In particular, adipocytes share a distinct cancer related characteristic (angiogenesis) suited to their ability for continuous proliferation and expansion. The data revealed that Resminostat reduced the gene expression of VEGF in Hep3B cells that was elevated by differentiated adipocytes CM. Moreover, the metastasis marker Vimentin that was increased at the protein level by adipocytes CM was also reduced by Resminostat. The RNA expression levels of several core oncogenes that are associated with excessive adipocytes including leptin, STAT3, TNFα and IL-10 were also targeted by Resminostat.

The utilisation of multiple therapeutic agents in cancer treatment is a useful anti-cancer strategy. While HDAC inhibitors have very promising results in both clinical and pre-clinical studies, they can also activate some off-target oncogenes by acetylation. Recent studies have demonstrated that HDAC inhibitors such as Resminostat have additive or synergistic activities in combination with other novel pharmaceutical agents and conventional chemotherapeutic agents for HCC treatment [15]. The results of this study confirmed the enhanced anti-proliferative activities of Resminostat when combined with other pharmaceutical agents including the FDA approved Sorafenib derivative SC-1 (10.27%), chemo-cytotoxic agents (Cisplatin (19.57%) and Doxorubicin (16.67%)) respectively.

The chromatin modifier EZH2 is a histone methyltransferase that is often over-expressed in many cancers including HCC [29]. In fact, EZH2 has been shown to co-operate with HDAC1 and suppress tumour suppressor microRNAs promoting metastasis in HCC [29 – 31]. Recent studies have demonstrated that HDAC 8 can directly interact with EZH2 to repress antagonists specifically targeting oncogenes thus promoting HCC onset and development in NAFLD patients [32]. We have shown that the RNA expression levels of HDAC 8 were significantly increased in Hep3B cells under the treatment of differentiated adipocytes CM. We next selected two novel EZH2 inhibitors DZNep and GSK343 to investigate their anti-cancer effects in combination with Resminostat against HCC. Recent studies reported that GSK343 is a direct EZH2 inhibitor and has a more potent anti-HCC effect than DZNep [33]. Our results showed that Resminostat increased the anti-proliferative effect of both EZH2 inhibitors by 11.76% (DZNep) and 21.61% (GSK343). Moreover, the combination of Resminostat and GSK343 enhanced the downregulation of EZH2 and STAT3 RNA expressions in Hep3B cells.

Epigenetic targeted therapies such as HDACi are ideal therapeutic strategies in cancer treatments. Nonetheless, their ability to silence or activate various genes can also lead to the induction of oncogenes, for example p65, leading to unwanted complications [19]. We selected the heat shock protein 90 inhibitor (HSP90i) 17-(allylamino)-17-demethoxygeldanamycin (17-AAG) as a secondary pharmaceutical agent that has been shown to specifically target inflammatory NF-κB activation [34]. This study revealed that the combination of Resminostat and 17-AAG had an enhanced anti-proliferative effect (31%) in HCC cells compared to single treatment when co-cultured with differentiated SGBS CM. In addition, we found that 17-AAG had a combined effect in the downregulation of HDAC1, HDAC2, the anti-apoptotic gene BCL-2 and tumour survival gene STAT3 in Hep3B cells in combination with Resminostat. Importantly, we demonstrated that 17-AAG enhanced the inhibitory function of Resminostat on ZFP64 gene expression in Hep3B cells. Interestingly, our data revealed that both Resminostat and 17-AAG abrogated the expression of HSP90 in Hep3B cells suggesting that Resminostat may also have a role in targeting imbalanced ER homeostasis. Importantly, 17-AAG not only downregulated the p65 nuclear protein levels in Hep3B cells, but also inhibited p65 at the RNA level under adipocyte CM or Resminostat treatment. While HDAC inhibitors can lead to acetylation of p65 potentially contributing to the inflammation in tumour microenvironment, the utilisation of 17-AAG as a combinatorial strategy may eliminate this pitfall of Resminostat, thus maximising the therapeutic outcome for HCC patients.

## CONCLUSION

The clinical outcome of HCC patients remains extremely poor. Recent clinical studies reported that a subgroup of advanced HCC patients may benefit more from Resminostat/Sorafenib combination treatment compared with Sorafenib alone. Intracellular lipid deposition has been widely agreed as a central pathologic characteristic in chronic liver disease patients associated with end stage hepatoma. Therefore, the findings of this study indicate that combinations of Resminostat/17-AAG as a therapeutic feature may provide a useful strategy in the clinical treatment of HCC.

## MATERIALS AND METHODS

### Cell lines and cell culture

Human hepatoma cell lines Hep3B and HepG2 cells were cultured in Minimum Essential Medium (EMEM Modified) supplemented with 10% (v/v) fetal bovine serum (FBS), 1% penicillin - streptomycin, and 2 mM L-glutamine at 37oC, 5% CO2 atmosphere. Liver cancer cell line Huh7 was cultured in Dulbecco's Modified Eagle's Medium (DMEM) supplemented with 10% (v/v) FBS, 1% penicillin - streptomycin, and 2 mM L-glutamine at 37oC, 5% CO2 atmosphere.

The human pre-adipocyte cell line was originally derived from the stromal cells fraction of subcutaneous adipose tissue of an infant with Simpson-Golabi-Behmel syndrome (SGBS) and was kindly given to us as a gift from Professor Wabitsch (University of Ulm, Germany). SGBS cell growth and differentiation were performed according to the standard protocol [35, 36]. It is worth noting that SGBS cell strain has been recently validated as a model for the *in vitro* study of obesity and cancer [37].

### Cell treatment

Liver cancer cell lines Hep3B, HepG2 and Huh7 were cultured and their conditioned mediums were collected. The human pre-adipocyte SGBS cells were differentiated and their conditioned mediums were collected.

Liver cancer cell lines Hep3B, HepG2 and Huh7 cells were treated with 30% of the CM from differentiated adipocytes, HDAC inhibitor Resminostat (80 nM), SC-1 (10 μM), cisplatin (10 μM), doxorubicin (1 μM), DZNep (20 μM), GSK343 (7 nM) and HSP90 inhibitor 17-AAG (50 nM) as designed (single or combination treatments). All concentrations were optimised with time points and doses.

### Global HDAC activity assay

Cells were cultured and treated as described. Cell lysates were obtained using CelLytic M Cell Lysis Reagent (C2978, Sigma Aldrich, Ireland). In brief, cells were washed with PBS and treated with CelLytic M Cell Lysis Reagent. The plate was incubated on a shaker at room temperature for 15 minutes. The lysed cells were then scraped from the plate and transferred to a 1.5 ml Eppendorf tube. The tube was centrifuged at high speed for 5 minutes and the supernatant was collected.

The global HDAC activity in cells was quantified using FluoroFire HDAC Activity Assay Kit (A00179, Molecutools, Ireland) according to the manufacturer's instruction. In brief, cell lysate samples, positive control (HeLa Nuclear Extract), negative control (HeLa Nuclear Extract with Trichostatin A solution) and blank (Assay Buffer) were added onto a black bottom 96-well plate. The plate was incubated at 37oC for 20 minutes and 50 μl HDAC Emerald Substrate working solution was added to each well. The plate was incubated at 37oC for 1 hour and fluorescence was read at Excitation 490 nm and Emission 525 nm.

### FluoroFire-Blue ProViaTox Assay (A00008, Molecutools, Ireland)

Cells (80 μL, 2000 cells) were seeded onto black bottom 96-well plates in triplicate. Cells were treated as previously described. The untreated cells were used as control. Wells containing medium without cells were used as reference. Following the treatment period, FluoroFire-Blue ProViaTox assay reagent (10 μL) was added onto each well and the plates were incubated at 37oC for between 2 and 4 hours. The fluorescence was read at 530 nm excitation and 590 nm emissions on SpectraMax M2. The proliferation of cells was represented as fold changes compared with controls.

### Caspase 3/7, 8, 9 Tetraplex Assay

The cleavage activities of apoptosis genes including Caspase 3/7, 8 and 9 were monitored in cells (untreated and treated) in real time by using FluoroFire Caspase 3/7,8,9 Tetraplex Assay Kit (A00030 Molecutools Ireland) according to manufacturer's protocol. Hep3B cells were seeded onto a black bottom 96 well plate at 20000 cells per well overnight and treated with SGBS CM and/or 17-AAG (50 nM) for a period of 24 to 72 hours. Assay buffer containing caspase substrates was added to each well during the treatment period to monitor real time caspase gene cleavage activities and measured in fluorescence of 535 nm Ex/620 nm EM (Caspase 3/7, Red fluorescence), 490 nm Ex/525 nm Em (Caspase 8, Green fluorescence) and 370 nm Ex/450 nm EM (Caspase 9, Blue fluorescence). Data is presented as RFU (relative fluorescence unit) and positively correlated with the cleavage activities of each caspase gene.

### RNA Extraction and real-time quantitative PCR (qRT-PCR) analysis

Total RNA was isolated using RNeasy Mini Kit (74104, Qiagen, UK) according to the manufacturer's instruction. The quality of isolated RNAs were validated by using 1% agarose gel and visualised under UV light using a Mini BisPro gel imaging system (DNR Bioimaging systems, Jerusalem, Israel) and Gel Capture Version 4.0.11.4 Software. The extracted total RNA was then reverse transcribed to complementary DNA (cDNA) on a GeneAmp PCR system 2400 (PERKIN ELMER) using Omniscript Reverse Transcription Kit (205111, Qiagen, UK) according to manufacturer's instruction.

In order to assess the expression levels of genes of interest, RT-qPCR was conducted using QuantiTect SYBR Green PCR Kit (204143, Qiagen, UK) according to the manufacturer's instruction. The β-Actin gene was used as a housekeeping gene to normalise Ct values and the primers of genes examined in this project were designed using qPrimerDepot and manufactured by Sigma Aldrich, Ireland (Primers’ sequences are listed in Table 1). Fold change in gene expression was analysed using standard protocol whereby the value of the controls was set to an arbitrary value of 1 [38].

### Nucleus Protein Extraction and active nucleus NFκB protein analysis

The nucleus proteins were extracted from the cells using NE-PER Nuclear and Cytoplasmic Extraction Reagents (78833, ThermoScientific, USA) according to the manufacturer's instruction. In brief, cells were cultured and treated in serum free medium in 6-well plates as described. Cells were harvested as pellet by using Trypsin-EDTA and washed with PBS. The supernatant was discarded and the cell pellet was treated with ice-cold Cytoplasmic Extraction Reagent I (CER I) followed by Ice-cold Cytoplasmic Extraction Reagent II (CER II). The supernatant containing cytoplasmic protein was transferred. The remaining insoluble pellet was re-suspended in ice-cold Nuclear Extraction Reagent (NER) for the isolation of nucleus protein. The quantity of the total nucleus protein was analysed using Bradford Assay according to the manufacturer's instruction [39].

The activated nucleus NFκB p65 protein was detected using Transcription Factor Kits for NFκB p50 and p65 (89858, ThermoScientific, USA) according to the manufacturer's instruction. In brief, working binding buffer (NFκB binding buffer, Poly dl.dC and Ultrapure water) was added to a 96-well NFκB Assay Plate coated with NFκB consensus duplex. The positive control (TNFα Activated HeLa Cell Nuclear Extract), negative control (blank well) and NE-PER Nuclear Extract samples were added onto the 96-well NFκB Assay Plate. The plate was sealed and incubated with mild agitation at room temperature for 1 hour. The plate was washed with 1X Wash Buffer for 3 times. The diluted NFκB p65 primary antibody was added to the plate and the plate was incubated without agitation at room temperature for 1 hour. The plate was washed with 1X Wash Buffer for 3 times. The diluted HRP-conjugated secondary antibody (100 μl) was added to the plate and the plate was incubated without agitation at room temperature for 1 hour. The plate was washed with 1X Wash Buffer for 4 times. The prepared substrate working solution (Luminol/Enhancer Solution and Stable Peroxide Solution) was added to the plate and the chemiluminescence was read at 570 nm on a spectrophotometer (SpectraMax M2, Molecular Devices).

### Statistical analysis

Experiments were performed in triplicates and validated three independent times. Data were analysed using PRISM 4.0 software package (GraphPad, San Diego, CA, USA). Results were expressed as the mean +/− standard deviation. The two-tailed paired/unpaired Student's t-test were used to analyse the statistical significance (* P < 0.05, ** P < 0.01, *** P < 0.001, **** P < 0.0001) of differences between mean values. One-way Anova test was used to analyse the statistical significance of differences between the means of two or more independent groups.

## SUPPLEMENTARY MATERIALS FIGURE AND TABLE


