# Supplementary material for: The therapeutic properties of resminostat for hepatocellular carcinoma
Source: Oncoscience. 2018 Jun 23;5(5-6):196–208. doi: 10.18632/oncoscience.420 (PMC6049311; doi:10.18632/oncoscience.420)
Supplement: Supplementary file 1 [file oncoscience-05-196-s001.pdf]

SUPPLEMENTARY MATERIALS

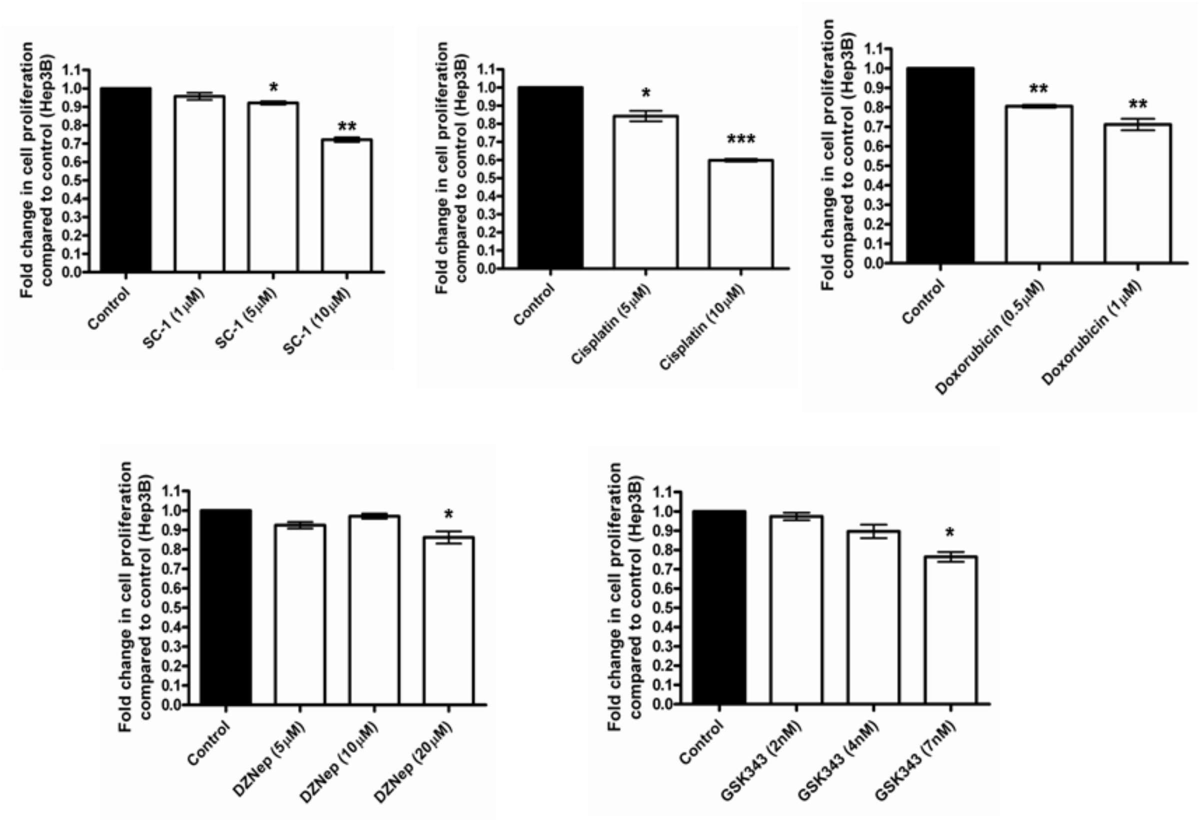

Supplementary Figure 1: Dose validation of secondary pharmaceutical agents in combination with Resminostat against HCC. Sc-1 (1 µM, 5 µM, 10 µM), Cisplatin (5 µM, 10 µM), Doxorubicin (0.5 µM, 1 µM), DZNep (5 µM, 10 µM, 20 µM) and GSK343 (2 nM, 4 nM, 7 nM) were used in dose validation in HCC cells examining their optimised dose for anti-proliferative effect (n = 3). Proliferation was measured with FluoroFire-Blue ProViaTox assay and presented as fold change compared to control (untreated cells).

Supplementary Table 1: Primer sequences

Gene Name	Sequence
β-Actin (ACTβ)	Forward: 5'-CCTTGCACATGCCGGAG-3'
	Reverse: 5'-GCACAGAGCCTCGCCTT-3'
HDAC 1	Forward: 5'-CATCTCCTCAGCATTGGCTT-3'
	Reverse: 5'-GACGGGGATGTTGGAAATTA-3'
HDAC 2	Forward: 5'-CAGCAAGTTATGGGTCATGC-3'
	Reverse: 5'-CCATGGCGTACAGTCAAGGA-3'
HDAC 3	Forward: 5'-GTTGTTCAGCTGGGTTGCTC-3'
	Reverse: 5'-GAGAGTCAGCCCCACCAATA-3'
HDAC 6	Forward: 5'-TCCAAGGCACATTGATGGTA-3'
	Reverse: 5'-CACAGTTCACCTTCGACCAG-3'
HDAC 8	Forward: 5'-CCAGCACATAATCAGGACCA-3'
	Reverse: 5'-ATTTTGGGAGGAGGAGGCTA-3'
Caspase 3	Forward: 5'-GAGTCCATTGATTCGCTTCC-3'
	Reverse: 5'-TCTGGTTTTCGGTGGGTG-3'
Caspase 7	Forward: 5'-TGCCCAGCTTTTCAAAATTC-3'
	Reverse: 5'-TCAGTGGATGCTAAGCCAGA-3'
Caspase 8	Forward: 5'-CGGAATGTAGTCCAGGCTCA-3'
	Reverse: 5'-GGTCACTTGAACCTTGGGAA-3'
Caspase 9	Forward: 5'-CACGGCAGAAGTTCACATTG-3'
	Reverse: 5'-ACACCCAGACCAGTGGACAT-3'
BCL-2	Forward: 5'-CGTACAGTTCCACAAAGGCA-3'
	Reverse: 5'-ATGTGTGTGGAGAGCGTCAA-3'
BAX (BCL2L4)	Forward: 5'-GGAGGAAGTCCAATGTCCAG-3'
	Reverse: 5'-TCTGACGGCAACTTCAACTG-3'
BIM (BCL2L11)	Forward: 5'-CCCTCCTTGCATAGTAAGCG-3'
	Reverse: 5'-CCAGGCCTTCAACCACTATC-3'
BAD (BCL2L8)	Forward: 5'-GGTAGGAGCTGTGGCGACT-3'
	Reverse: 5'-GCTCCGGCAAGCATCAT-3'
Cyclin D1 (CCND1)	Forward: 5'-TGAGGCGGTAGTAGGACAGG-3'
	Reverse: 5'-GACCTTCGTTGCCCTCTGT-3'
P21 (CDKN1A)	Forward: 5'-GCCATTAGCGCATCACAGT-3'
	Reverse: 5'-ACCGAGGCACTCAGAGGAG-3'
P27 (CDKN1B)	Forward: 5'-ACAGGATGTCCATTCCATGA-3'
	Reverse: 5'-GGCCTCAGAAGACGTCAAAC-3'
TNFα	Forward: 5'-AGATGATCTGACTGCCTGGG-3'
	Reverse: 5'-CAGCCTCTTCTCCTTCCTGA-3'
VEGF	Forward: 5'-CACACAGGATGGCTTGAAGA-3'
	Reverse: 5'-AGGGCAGAATCATCACGAAG-3'
Leptin	Forward: 5'-GACTTTTTGGATGGGCACAG-3'
	Reverse: 5'-GTAGGAATCGCAGCGCC-3'
IL-10	Forward: 5'-GCCACCCTGATGTCTCAGTT-3'
	Reverse: 5'-GTGGAGCAGGTGAAGAATGC-3'
STAT3	Forward: 5'-GGCCATCCTGCTAAAATCAG-3'
	Reverse: 5'-GTCTCTCCCCCTCGGCT-3'
P16 (CDKN2A)	Forward: 5'-GGTCGGGTGAGAGTGGC-3'
	Reverse: 5'-CCCAACGCACCGAATAGTTA-3'
EZH2	Forward: 5'-GCCAACAAACTGGTCCCTT-3'
	Reverse: 5'-GGACTCAGAAGGCAGTGGAG-3'
ZFP64	Forward: 5'-GTGACTTGGCAGGGAGAGAG-3'
	Reverse: 5'-ACCACCAGAACCATCACCTC-3'
HSP90	Forward: 5'-CAATGACATCAACTGGGCAA-3'
	Reverse: 5'-CTGTGCCGTTGGTCCTGT-3'
P65 (RELA)	Forward: 5'-TTTCTCCTCAATCCGGTGAC-3'
	Reverse: 5'-ACCCCTCCCTACGCAGAC-3'
